# Utility of Radiological Follow Up of Main-Duct Intraductal Papillary Mucinous Neoplasms and Mixed-Type Intraductal Papillary Mucinous Neoplasms

**DOI:** 10.3390/biomedicines12071437

**Published:** 2024-06-27

**Authors:** Roie Tzadok, Rivka Kessner, Einat Ritter, Asaf Aizic, Hila Yashar, Sapir Lazar, Yuval Katz, Zur Ronen-Amsalem, Arthur Chernomorets, Oren Shibolet, Dana Ben-Ami Shor

**Affiliations:** 1 Department of Gastroenterology and Liver Diseases, Tel Aviv Sourasky Medical Center, Faculty of Medicine, Tel Aviv University, Tel Aviv 69978, Israel; roie.tzadok@gmail.com (R.T.); einatri@tlvmc.gov.il (E.R.);; 2 Department of Radiology, Tel Aviv Sourasky Medical Center, Faculty of Medicine, Tel Aviv University, Tel Aviv 69978, Israel; 3 Department of Pathology, Tel Aviv Sourasky Medical Center, Faculty of Medicine, Tel Aviv University, Tel Aviv 69978, Israel

**Keywords:** intraductal papillary mucinous neoplasm, pancreatic ductal adenocarcinoma, main pancreatic duct, magnetic resonance cholangiopancreatography

## Abstract

Background: Intraductal papillary mucinous neoplasms (IPMNs) have the potential to evolve into pancreatic adenocarcinoma (PDAC). While main-duct IPMNs (MD-IPMNs), involving the main pancreatic duct (MPD), are less common than side-branch IPMNs (SB-IPMNs) or mixed-type IPMNs (mixed-IPMNs), their malignant transformation potential is far greater. Controversy exists between different guidelines in terms of recommended management strategies. This study was aimed at assessing the utility of the radiological follow up of MD-IPMNs and mixed-type IPMNs, including prevalence of worrisome radiological findings as well as clinical and laboratory parameters, and their correlation with the development of progression or pancreatic adenocarcinoma. Methods: Eighty-four patients with MD-IPMNs or mixed-type IPMNs who underwent at least one magnetic resonance cholangiopancreatography (MRCP) were included. Clinical and laboratory data were obtained retrospectively. A cross-sectional analysis was carried out to establish clinical and laboratory parameters associated with development of PDAC. A retrospective cohort analysis was performed on 44 patients who had at least six months of follow up, trying to identify factors correlating with worrisome radiological features. Results: Nine cases (10.7%) of PDAC were recorded in this cohort. The laboratory and imaging factors associated with cyst size progression greater than 5 mm during follow up were elevated alanine transaminase (ALT) levels, the maximal cyst size, and the MPD diameter. Cross-sectional analysis indicated that PDAC was associated with nausea (*p* = 0.01), as well as increased levels of aspartate aminotransferase (AST) (*p* = 0.05), gamma glutamyl transpeptidase (GGT) (*p* = 0.01), and alkaline phosphatase (ALP) (*p* = 0.01). Conclusions: Elevated levels of liver enzymes were associated with IPMN progression and, subsequently, the development of PDAC. ALT levels, maximal cyst size, and MPD diameter are associated with the progression of cyst size. These data may aid in risk-stratifying patients when determining the follow up approach for IPMNs.

## 1. Introduction

Pancreatic cysts are frequently diagnosed incidentally during abdominal imaging conducted for unrelated reasons. Their prevalence increases with age and has been previously estimated to be as high as 13.5% of all abdominal MRI tests performed [1]. Intraductal papillary mucinous neoplasms (IPMNs), first described by Ohashi et al. in 1982 [2], represent the most common type of cystic pancreatic lesions, accounting for up to 70% of cases [3]. Since 1996, the World Health Organization has recognized IPMNs as a distinct entity and incorporated them into the International Histological Classification of tumors [4].

Although all IPMNs are well-established precancerous lesions in the pancreas, they are classified based on their location and involvement within the pancreatic duct, which can be categorized as either the main duct, branch duct, or a mixed type combining both [5,6,7]. This anatomical classification also carries prognostic significance concerning the potential for malignant transformation into adenocarcinoma. Main-duct IPMNs (MD-IPMNs), which involve the main pancreatic duct (MPD), are less common than side-branch IPMNs (SB-IPMNs) or mixed-type IPMNs (mixed-IPMNs). However, MD-IPMNs have a considerably higher potential for malignant transformation [8], with the reported rates of high-grade dysplasia or pancreatic cancer found in resected specimens being as high as 68% [9].

With the growing use of abdominal imaging and advancements in imaging techniques, the diagnosis of pancreatic cysts is on the rise. While some of these cysts have the potential to progress into pancreatic ductal adenocarcinomas (PDAC), highlighting the importance of early detection through IPMN surveillance [10], it has been observed that the increased incidence of diagnosis is not necessarily accompanied by a corresponding increase in pancreatic adenocarcinoma-related deaths, further emphasizing the question of the clinical relevance of these radiologic findings. In fact, updated Surveillance, Epidemiology, and End Results Program (SEER) data indicate that pancreatic cancer-related mortality has remained relatively stable over the past few decades [11,12]. This discrepancy may suggest a problem of overdiagnosis, presenting clinicians with the challenge of identifying patients who truly require further investigation and follow up while attempting to minimize unnecessary diagnostic tests.

The precise risk of cystic pancreatic lesions transforming into malignant tumors is variable, not entirely clear, and often characterized by contradictory data. While it is important to note that not all pancreatic cystic lesions possess malignant potential, those that do were previously estimated to have an average transformation prevalence rate of 2.5%. This rate varies, increasing with age and reaching as high as 8.7%, with a potential lifetime risk of up to 25%, as reported in some studies [13,14]. Alternatively, the yearly risk has been approximated at 0.24% [15]. However, previous studies have suggested significantly higher transformation rates, with some cohorts reporting rates as high as 40% [16,17].

As detection rates continue to rise, considering the relatively low risk of malignant transformation along with the costs associated with cyst surveillance on the one hand and the potentially fatal effects of PDAC and the morbidity linked to pancreatic resection surgeries on the other, it has become imperative to establish criteria for cyst risk stratification and surveillance based on radiological characteristics. Opting for conservative management, whenever feasible, empowers both the clinician and the patient to avoid unnecessary pancreatic resections, which are known to be associated with high rates of morbidity and mortality [15].

Current guidelines rely on identifying worrisome radiological features and high-risk stigmata of IPMNs to determine the most appropriate management strategies, including surgical resection, endoscopic ultrasound (EUS) with fine-needle aspiration (FNA), and long-term radiological follow-up. Parameters such as cyst size, cyst growth rate, MPD diameter, the presence of lymphadenopathy, the radiological observation of an enhancing mural nodule, as well as laboratory indicators such as carbohydrate-antigen 19-9 (CA 19-9), are all considered when making decisions regarding the optimal management approach. However, there is ongoing controversy among the different guidelines regarding the recommended mode, intervals, and duration of follow up, which has resulted in a lack of consensus in the clinical practice of patients with IPMNs [8,18,19,20].

This study aimed to identify radiologic, clinical, and laboratory predictors for MD-IP and mixed-type IPMN progression, including assessing the prevalence of worrisome radiological findings and their association with the development or progression of PDAC.

## 2. Materials and Methods

### 2.1. Study Population

A total of 3936 magnetic resonance cholangiopancreatography (MRCP) examinations were conducted at our center between the years 2011 and 2021. The examinations were performed on a 1.5 T or 3.0 T Siemens MRI systems (Avanto^®^, Skyra^®^, Aera^®^, or VIDA^®^; Siemens Healthineers, Erlangen, Germany) with an abdominal radiofrequency coil. Our pancreatic MRI with MRCP protocol included multiple T2 sequences: an axial half Fourier single-shot turbo spin-echo (HASTE) T2 sequence of the entire upper abdomen, in free breathing with a slice thickness of 5 mm; a coronal HASTE T2 sequence in free breathing with a slice thickness of 5 mm; an axial HASTE T2 sequence in free breathing, with inversion recovery and a slice thickness of 5 mm; an axial breath-hold HASTE T2 sequence with a small field of view (focused on the pancreas) and a slice thickness of 3 mm; and 3 scans of a coronal breath-hold HASTE T2 sequence with a slice thickness of 3 mm—true coronal, left anterior oblique and right anterior oblique. The MRCP portion included 2 sequences of 3D respiratory triggered coronal single-slab 3D turbo spin-echo T2, with and without minimal intensity projection. Diffusion-weighted images were acquired in free breathing with a slice thickness of 5 mm and b-values of 50, 400, and 800 s/mm^2^. Out-of-phase and in-phase gradient-echo scans of the upper abdomen were acquired on the axial plane with a slice thickness of 3 mm. The last part of the examination included several axial volumetric interpolated breath-hold (VIBE) T1 scans of the upper abdomen, with slice thicknesses of 3 mm, before and after intravenous contrast administration. We used 0.2 mL/kg gadoterate meglumine (Dotarem, Guerbet, Villepinte, France) as the contrast material for pancreatic MRI. The post-contrast scans were performed at 25 s, 55 s, 90 s, 3 min, and 5 min after contrast administration.

Among these patients, a previous documented radiological diagnosis of pancreatic IPMNs was found in 1603 cases. The study population consisted of 84 patients aged 18 years and above, all of whom had undergone at least one MRCP and had a prior documented radiological diagnosis of either MD-IPMNs or mixed-type IPMNs. Excluded from the study were pregnant women, individuals under 18 years old, and patients diagnosed with SB-IPMNs or pancreatic cysts other than IPMNs. 

The reports of 170 MRCPs were reviewed, with an average of 2 examinations per patient. For each patient, assessments were made regarding cyst size, cyst location, and the presence of worrisome features and/or high-risk stigmata of IPMNs, including the presence of an intra-cystic mass, pancreatic duct dilation, and lymphadenopathy [18]. Most of the reports included all of the parameters. In 6 cases, the initial report was not complete, and a review of the examinations was performed by an abdominal imaging certified radiologist (RK) from the radiology department of our center.

Clinical and laboratory data were collected retrospectively from medical records, including the referral notes, clinical records from the gastroenterology and surgical departments, the EUS examination report, the cytology/pathology report, and laboratory results. The data encompassed past medical history including previous pancreatitis, prior extra-pancreatic malignancies, liver disease, weight, height, Body Mass Index (BMI), history of smoking and alcohol abuse, family history of PDAC, blood tests performed up to 6 months before or after each MRCP (complete blood count, blood chemistry tests including liver enzymes, blood tumor markers such as carcinoembryonic antigen (CEA) levels in ng/mL, and CA19-9 levels in U/mL), patient-reported gastrointestinal symptoms, EUS follow-up, and available pathology reports. All data were retrieved from our institutional computerized database. All data parameters collected are depicted in Appendix A.

### 2.2. Statistical Analysis

A cross-sectional analysis aimed at identifying clinical and laboratory parameters associated with the development of PDAC was carried out. Subsequently, a retrospective cohort analysis was conducted on 44 patients from the original study population who had at least six months of follow-up. The objective was to identify factors correlating with worrisome radiological features based on the revised Fukuoka guidelines for the management of IPMNs of the pancreas [18].

Categorical variables were presented as frequencies and percentages, while the distribution of continuous variables was assessed using histograms. Given that all continuous variables did not follow a normal distribution, they were reported as medians and interquartile ranges (IQRs). Age and age at MRCP were presented as means and standard deviations. 

To examine the association between categorical variables and the presence of PDAC at the initial evaluation, Fischer’s exact test was employed. Meanwhile, the Mann–Whitney test was used to assess the association of continuous variables. Events occurring during the follow-up period were illustrated using Kaplan–Meier curves. All statistical tests were two-sided, and *p*-values under 0.05 were considered statistically significant. 

For our statistical analyses, we utilized SPSS software (IBM Corp. Released 2021. IBM SPSS Statistics for Windows, Version 28.0., IBM Corp., Armonk, NY, USA).

## 3. Results

A total of eighty-four patients, comprising forty males and forty-four females, with a mean age of 74 ± 9.03 years, were included in the cohort study. Sixty-nine (82.1%) presented with mixed-type IPMNs, whereas 15 (17.9%) presented with MD-IPMNs. The mean radiological follow-up time was 26.51 ± 18.75 months, and the median time from the initial diagnosis of a radiological pancreatic finding to the first MRCP being performed was found to be 6.3 months (IQR 2.4–19.2 months). A comprehensive description of the patients’ baseline characteristics is provided in Table 1.

**Figure 1:**
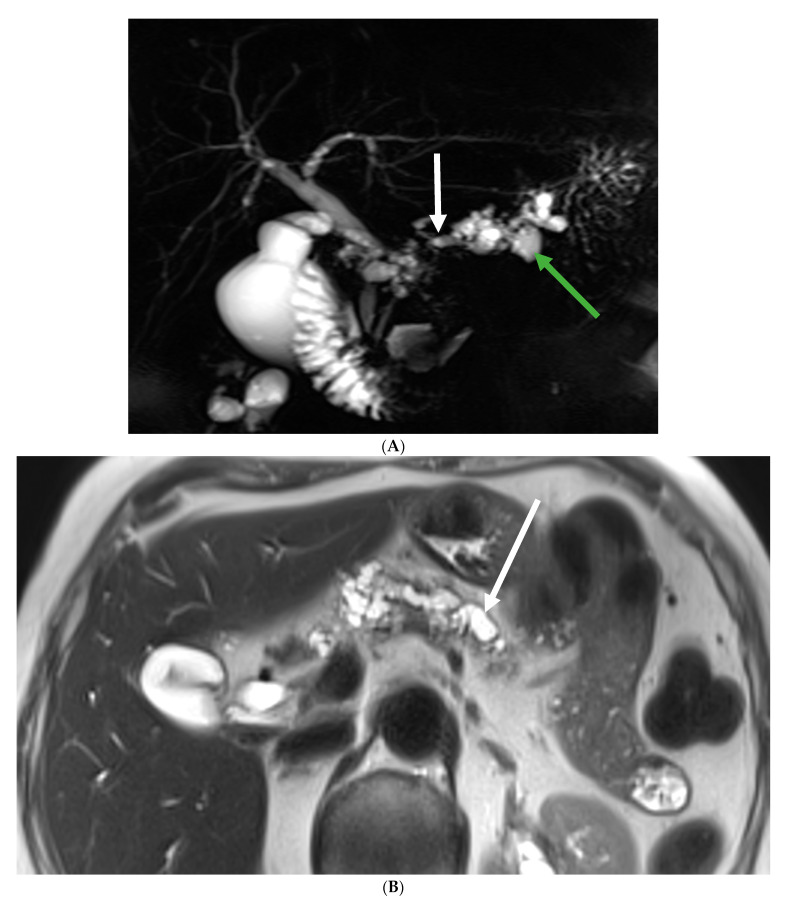

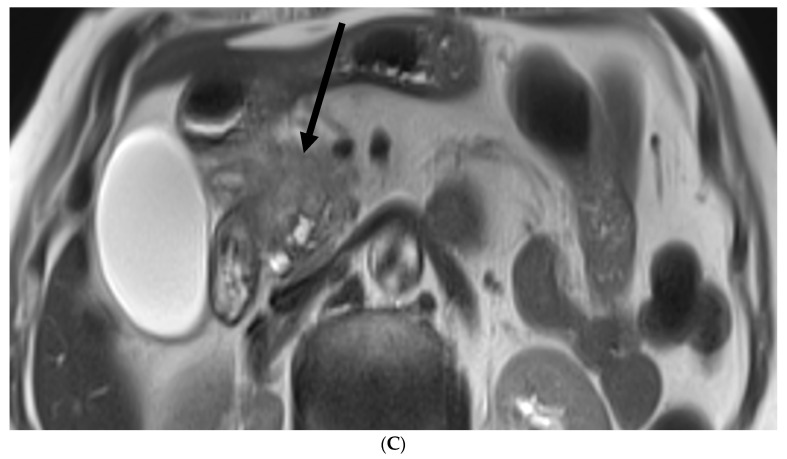
Eighty-year-old female diagnosed with PDAC at first MRCP. (**A**) Several dilatations of the main pancreatic duct (white arrow) and multiple pancreatic cystic lesions (green arrow); (**B**) several dilatations of the main pancreatic duct (white arrow) and multiple pancreatic cystic lesions; (**C**) a mass with an intermediate signal and ill-defined borders is noted in the head of the pancreas (black arrow).

**Figure 2:**
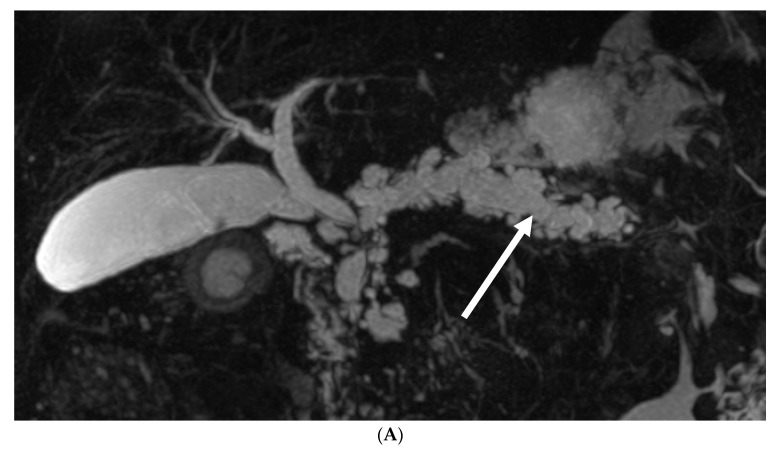

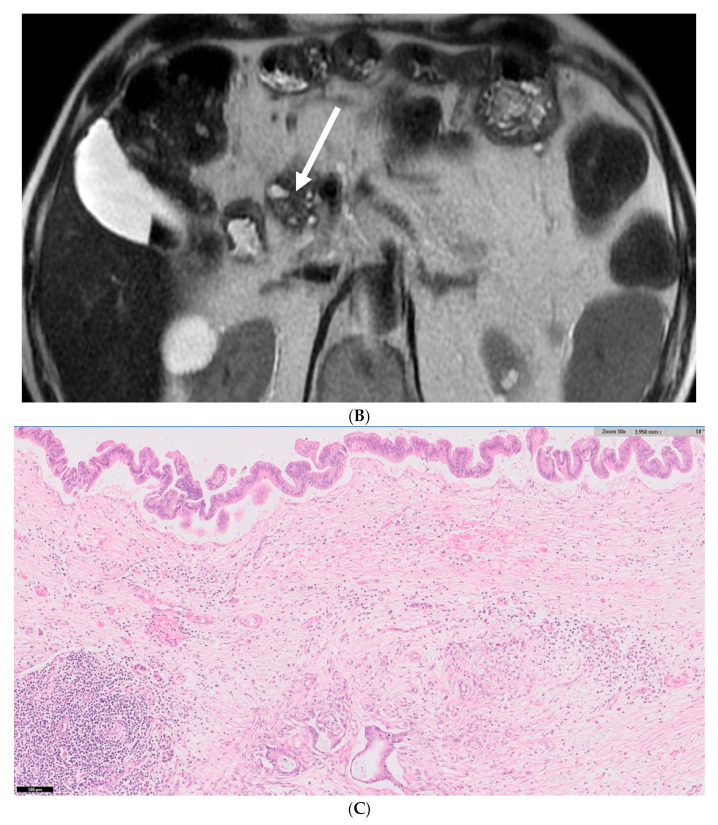
Seventy-six-year-old male with dilatation of the main pancreatic duct. (**A**) Dilatation of the main pancreatic duct (arrow) and multiple side branches on MRCP; (**B**) an ill-defined heterogeneous mass (arrow) is seen in the head of the pancreas on T2; (**C**) an IPMN is seen on the upper side of the image, while an invasive adenocarcinoma is seen of the lower side of the image.

Within this patient cohort, a total of nine cases (10.7%) of PDAC were meticulously recorded. Notably, five of these cases (four mixed-IPMNs and one MD-IPM) were promptly diagnosed at the time of presentation, with three occurring in male patients and two in female patients. MRCP imaging and histology specimens of two of these cases are presented in Figure 1 and Figure 2.

**Figure 3:**
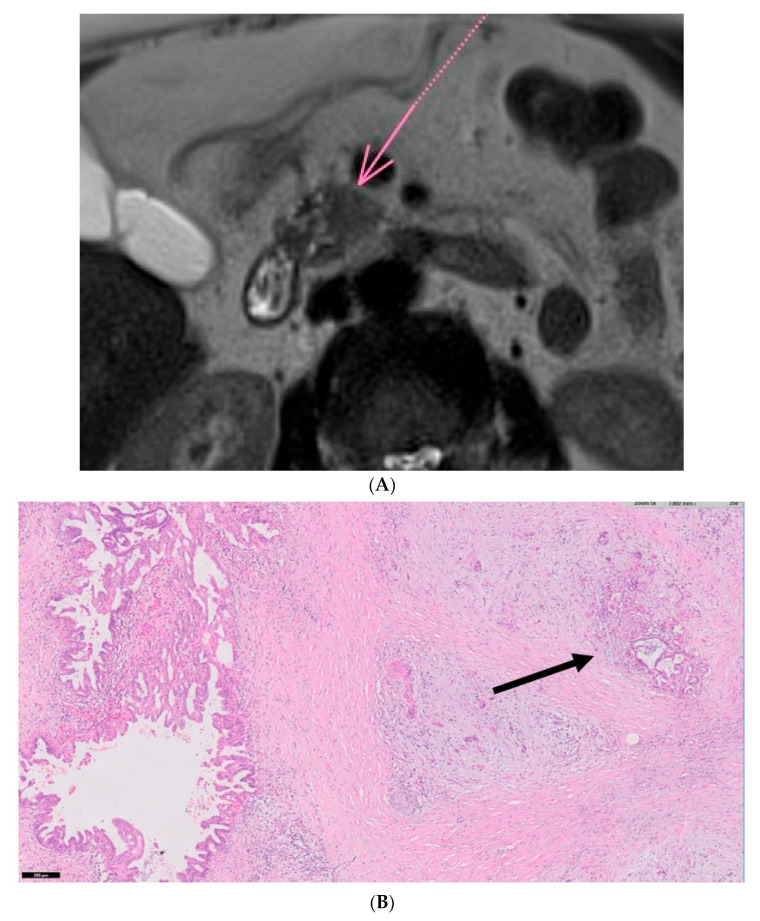
Eighty-four-year-old female with PDAC developed during follow-up. (**A**) T2-weighted image of PDAC. The lesion is marked with an arrow. (**B**) (H&E, ×5): An IPMN is seen on the left side of the image, while an invasive adenocarcinoma with perineural invasion (arrow) is seen on the right side of the image.

Four more cases of PDAC emerged during the follow-up period. All of them were females: one was an 83-year-old female, diagnosed with a mixed-IPMN, who was asymptomatic, and presented with elevated alanine transaminase (ALT). A second patient was an 81-year-old female diagnosed with a mixed-IPMN, with a family history of PDAC who was also asymptomatic, and also presented with elevated ALT. The third was an 83-year-old female diagnosed with MD-IPMN, with a family history of PDAC, who presented with nausea and abdominal bloating. The fourth was a 76-year-old female diagnosed with a mixed-IPMN, who was asymptomatic and presented with elevated alkaline phosphatase (ALKP). Imaging and histology of one of these cases are presented in Figure 3.

**Figure 4:**
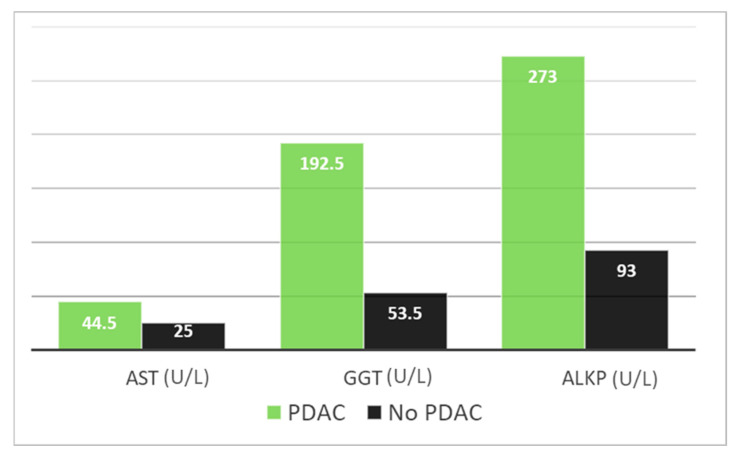
Median liver enzyme levels among patients developing PDAC compared to those who did not. ALKP—alkaline phosphatase; AST—aspartate aminotransferase; GGT—gamma glutamyl transpeptidase; PDAC—pancreatic ductal adenocarcinoma. Legend: Median AST (U/L), GGT (U/L), and ALKP (IU/L) among patients developing PDAC in a cross-sectional analysis versus those who did not. Values in the PDAC group were significantly higher (*p*-value 0.05 for AST, 0.01 for GGT and ALKP).

A cross-sectional analysis was carried out to delve deeper into the factors associated with the development of PDAC. It was discerned that the emergence of PDAC was statistically linked to patients’ complaints of nausea (*p* = 0.01). Moreover, elevated levels of certain biochemical markers, including aspartate aminotransferase (AST), were noted in the pancreatic cancer group. Specifically, the median AST level in the pancreatic cancer group was 44.5 U/L, with an IQR of 26–62 U/L, in contrast to the cancer-free group, which exhibited a median AST level of 25 U/L, with an IQR of 19–37.25 U/L (*p* = 0.05). Furthermore, gamma glutamyl transpeptidase (GGT) levels were substantially elevated in the pancreatic cancer group, with a median value of 192.5 U/L compared to 53.5 U/L in the cancer-free group, with an IQR of 102–1681 U/L vs. 20–73 U/L, respectively (*p* = 0.01). A similar trend was observed with ALKP, where the pancreatic cancer group displayed a median value of 273 IU/L, while the cancer-free group had a median of 93 IU/L, with an IQR of 162–350 IU/L vs. 65–134 IU/L, respectively (*p* = 0.01). These findings are presented in Figure 4.

To further elucidate the factors contributing to the development of worrisome radiological features, a retrospective analysis was conducted. This analysis involved forty-four patients (20 male, 24 female) with a mean age of 72.74 ± 9.65 years who were subjected to a minimum of 6 months of follow up between their first and final MRCP examinations. The primary objective was to identify factors that correlated with the emergence of worrisome radiological features, as precisely defined by the revised Fukuoka guidelines. These worrisome features encompassed clinically apparent pancreatitis; a cyst size ≥ 3 cm; an enhancing mural nodule size > 5 mm; thickened or enhanced cyst walls; the main pancreatic duct (MPD) diameter measuring 5–9 mm; abrupt changes in the pancreatic duct caliber; lymphadenopathy; elevated CA 19–9 levels; and cyst size progression of 5 mm or more over a 2-year period [18].

**Figure 5:**
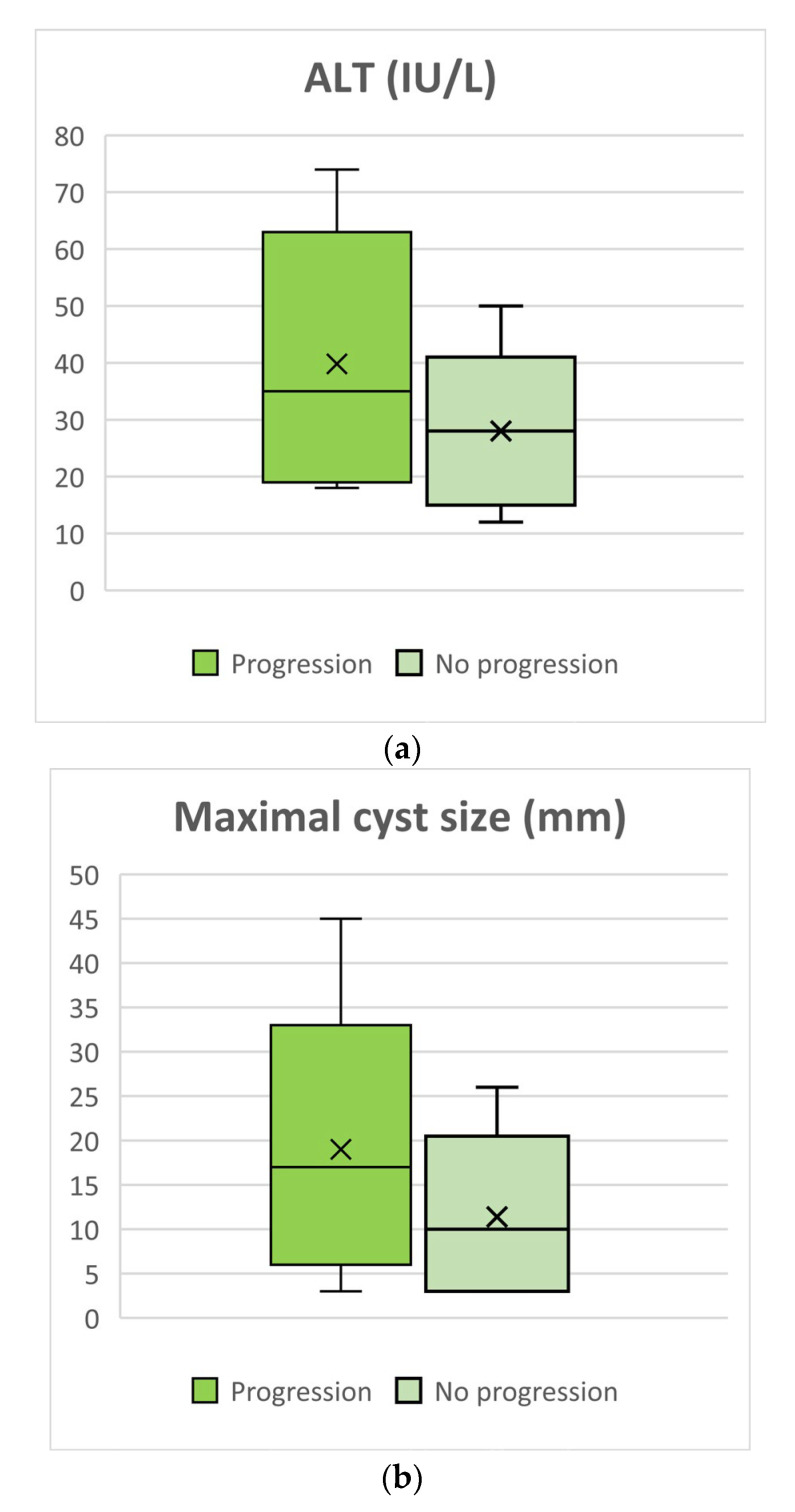

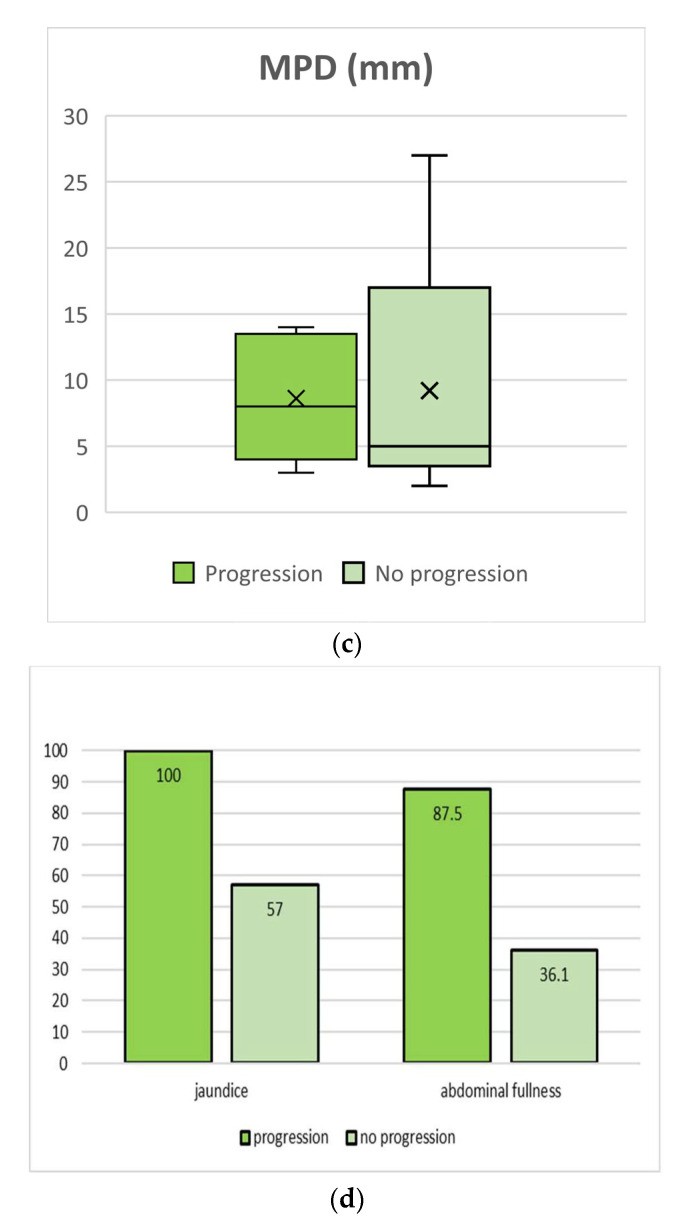
Factors associated with maximal cyst progression ≥ 5 mm. (**a**) ALT values in patients with maximal cyst progression ≥5 mm over the follow-up period were significantly higher than in patients with more stable IPMNs (*p* = 0.007). (**b**) Maximal cyst size in patients with cyst progression ≥ 5 mm over the follow-up period was significantly higher than in patients with more stable IPMNs (*p* = 0.02). (**c**) MPD diameter in patients with cyst progression ≥ 5 mm over the follow-up period was significantly higher than in patients with more stable IPMNs (*p* = 0.01). (**d**) The prevalence of jaundice and complaints of abdominal bloating was significantly higher among patients with cyst progression ≥5 mm over the follow-up period (*p* = 0.04 for jaundice and 0.008 for bloating).

Maximal cyst size progression in consecutive imaging tests was followed up, and the time taken to progress to 3 mm and 5 mm in size was measured. Upon meticulous analysis, several factors were identified to be closely associated with cyst progression to sizes greater than 5 mm during the follow-up period. Notably, ALT levels exhibited a significant difference between the stable cyst size group and the progression group. Specifically, the stable cyst size group displayed a median ALT level of 28 IU/L, with an IQR of 18–32, while the progression group exhibited a median ALT level of 35 IU/L, with an IQR of 20–52 IU/L (*p* = 0.007). These data are presented in Figure 5a. The maximal cyst size also played a pivotal role, with the progression group showing a median maximal cyst size of 17 mm (IQR: 9–21 mm), whereas the stable cyst size group had a median maximal cyst size of 10 mm (IQR: 3–15 mm), further emphasizing the significance of cyst size in the context of progression (*p* = 0.02). These data are presented in Figure 5b. Furthermore, the baseline MPD diameter was examined, revealing a median MPD diameter of 8 mm in the progression group compared to 5 mm in the stable cyst size group (IQR: 5–13 mm vs. 5–7 mm, respectively *p* = 0.01). These data are presented in Figure 5c. Lastly, it was noted that jaundice (*p* = 0.04) and abdominal bloating (*p* = 0.008) were significant clinical factors associated with cyst progression greater than 5 mm during follow up. These data are presented in Figure 5d.

Among seven patients with IPMNs and PDAC who presented with elevated cholestatic and/or hepatocellular liver enzymes, four also had additional IPMN lesions in the pancreas, while one patient had a cystic lesion in the head, and three others had them scattered along the parenchyma. The mean MPD size in these patients was 9 mm, with four of them showing evidence of common bile duct compression by the dilated MPD. None of the patients displayed any evidence of liver metastases.

## 4. Discussion

This study aimed to assess the utility of current guidelines regarding the follow up of MD-IPMNs and mixed-IPMNs, while correlating worrisome radiological features with PDAC development and progression. Liver enzyme levels were found to be linked both to the development of pancreatic adenocarcinoma and accelerated cyst progression (over 5 mm during the follow-up period).

The issue of cyst progression as a predictor of malignant potential is of special importance, as the current resection criteria for IPMNs set by the Fukuoka guidelines do not have a satisfying predictive value for high-risk patients [21]. As recently shown by Overbeek et al., these patients, who have a ≥10% lifetime PDAC risk, have an even higher cumulative incidence of IPMNs, accelerated growth rate, and higher malignancy risk, necessitating the adoption of better criteria to improve risk stratification [22].

Gomez et al. previously demonstrated that average ALT levels were more common among patients with IPMNs and PDAC rather than patients with completely benign pancreatic lesions, such as serous cystadenoma, a finding which was only hypothesized in later works to be associated with related liver involvement of the neoplastic process. In their study, the authors found a correlation between liver function abnormalities, such as elevated serum ALT and ALKP, and pre-malignant or malignant pancreatic cystic lesions, similar to the way in which radiological biliary obstruction or dilatation is associated with these lesions. This correlation was particularly prominent in cases where the cystic lesions measured less than 2.5 cm and were located in the head, neck, or uncinate process of the pancreas. Given the increased complexity of managing small cystic lesions, these straightforward and routine laboratory markers may serve as valuable guides for clinicians [23].

In a similar fashion, previous studies conducted by Zhang et al. and He et al. demonstrated a negative correlation between transaminases and prognosis in patients with an established diagnosis of PDAC [24,25], a correlation that may be attributed to liver invasion by tumor cells. Elevated ALKP, as a surrogate marker of biliary obstruction, was also shown to be of predictive value [23]. The relationship between elevated ALKP and the progression of mixed-IPMNs into PDAC was further demonstrated in another study by Roch et al. [10]: in their large prospective database, they identified serum CA 19-9 and elevated ALKP levels as the laboratory markers that exhibited the highest predictive value for invasiveness. In a prior study conducted by the same research group, it was observed that elevated glucose levels and increased ALKP levels were correlated with malignancy and were consistent with the presence of jaundice and diabetes mellitus [26].

This is the first study, to our knowledge, to indicate a correlation between IPMNs progressing to PDAC and elevated transaminases. Although one possible hypothesis is that patients who demonstrate elevated liver enzymes in retrospective analysis may have had an undiagnosed liver-spreading tumor nidus at baseline, larger prospective studies are needed not only to establish the causation but also in an attempt to validate transaminases cut-off values to warrant a more thorough investigation of IPMNs.

GGT is an enzyme not specific to the liver, and is also found in the kidneys, pancreas, heart, and brain. While it serves as a sensitive yet non-specific marker for liver dysfunction [27], it is a known inducer of oxidative stress and inflammation, which were hypothesized to be involved in cancer development and progression. Increased GGT levels have been demonstrated to be involved in several carcinogenic processes [28,29,30]. A previous work by Xiao et al. showed elevated serum GGT levels to be associated with decreased overall survival in PDAC patients [27]. To our knowledge, however, no previous data exist regarding GGT as a prognostic factor for IPMNs’ development to PDAC. Nevertheless, inflammatory mechanisms were shown to play a part in IPMN progression to adenocarcinoma: for example, elevated levels of the inflammatory cytokine IL-1β were found in fluid aspirated from IPMNs harboring high-grade dysplasia or carcinoma (in comparison to low-grade dysplasia) [31], and increased neutrophilic infiltration was noted in IPMNs transforming into cancer [32].

Current guidelines regarding IPMN surveillance stratify clinical, laboratory, and imaging features according to their relative risk for PDAC development (i.e., “high risk stigmata” and “worrisome features” according to the revised Fukuoka guidelines, but also parallel recommendations by the American College of Gastroenterology) [8,15,18]. Liver enzymes are not included as a prognostic feature in any of them, and while utilizing them will require further research and validation, they may also aid as a clinical tool in deciding whether to refer a patient to a pancreatic resection surgery or continue radiological follow up.

Our cohort included 15 cases of MD-IPMNs. As mentioned, these neoplasms harbor a greater risk of malignant transformation [8]. In a previously published study, Masaki et al. regarded the issue of MD-IPMN as an absolute indication for pancreatic surgery. Twenty-nine cases of MD-IPMNs with a mural nodule ≥ 5 mm resulting in surgery were reviewed retrospectively with a revision of the pathologic report. MPD diameter ≥ 12 mm was suggestive of malignancy [33]. Uehara et al. showed that the cut-off value for increased risk of malignancy was 15 mm in a retrospective cohort of 58 MD-IPMN patients [34]. While our cohort was not large enough to establish such cut-off values with sufficient sensitivity and specificity, we showed that among patients whose cysts progressed, the average MPD values were also significantly higher. However, given that we only addressed MPD progression and not an established diagnosis of PDAC, the mean MPD values in our cohort were lower.

The issue of MPD diameter as a prognostic sign of malignancy is subject to open debate, and there is lack of consistency between various guidelines in clinical practice: these guidelines primarily rely on retrospective analyses of surgical cases, which can introduce bias, and the precise malignancy risk associated with various MPD distention cut-offs remains uncertain. As emphasized by Kim et al., the 2017 revised international consensus guidelines and the 2018 European guidelines state that all surgically fit patients with MD-IPMNs and mixed-IPMNs with a high-risk feature of an MPD diameter of 10 mm or more should be recommended to undergo surgical resection [35]. However, those guidelines were, at least in part, based on evidence showing that over a cut-off of 5 to 7 mm, there is increased risk of IPMNs harboring high-grade dysplasia or malignancy. Yet, data regarding risk in the MPD diameter range of 5–9 mm re limited. Similarly, Sugimoto et al., revising the 2012 version of the international consensus guidelines for the management of IPMNs [36], concluded that an MPD diameter cut-off of 7.2 mm or greater as one of the high-risk stigmata had a higher sensitivity, accuracy, and negative predictive value for malignancy compared to the accepted 10 mm [36]. In contrast, Ceppa et al. conducted an evaluation of patients with invasive PDAC arising from various types of IPMNs. Their findings revealed that none of the so-called IPMN high-risk stigmata, such as the MPD diameter or the presence of a solid component, could predict overall survival. Factors like a family history of pancreatic cancer and lymph node involvement were more significant in this regard when compared to other variables [37].

Our study has several clear limitations: first, its retrospective nature, limiting the data quality and correlation levels which can be proven, as well as introducing biases related to data collection and patient selection. Second, our small cohort size, resulting mainly from the fact that this study was limited to MD-IPMNs and mixed-type IPMNs, which are much less common than side-branch IPMNs, might limit the generalizability of the findings. Third, our short follow-up time, and consequently the cross-sectional nature of our study, did not enable us to establish a cause-and-effect relationship between certain IPMN characteristics and their natural evolution.

In conclusion, this study shows that elevated liver enzymes are associated with IPMN progression and PDAC development. It is, to our best knowledge, the first to establish liver enzymes not only as a marker correlated with proven PDAC but also as a risk factor of IPMNs’ malignant transformation over time. Further prospective studies, including larger cohorts of main, mixed, and side-branch IPMNs, are necessary to strengthen and establish our findings and validate their clinical significance.

## Figures and Tables

**Table 1:** Baseline clinical characteristics of study population (n = 84).

Gender, male (%)	40 (47.6)	IPMN type	
Age at IPMN diagnosis (mean, SD)	72.4, 9.3	Mixed-IPMN	65 (82.1)
Age at first MRCP (mean, SD)	74.0, 9.0	MD-IPMN	15 (17.9)
Time (months) from diagnosis to first MRCP (median, IQR)	6.3, 2.4–19.2	Pancreatic cyst location	
DM (%)	35 (41.7)	Head	23 (27.4)
BMI		Uncinate	7 (8.4)
18.5>	3 (3.6)	Neck	1 (1.2)
24.9–18.5	15 (17.9)	Body	18 (21.4)
29.9–25	44 (52.4)	Tail	6 (7.1)
30<	22 (26.2)	Multiple location	29 (34.5)
Dyslipidemia (%)	48 (57.1)		
Smoking (past/present) (%)	29 (34.5)		
Ethanol use (%)	1 (1)		
Previous pancreatitis (%)	6 (7.1)		
Liver diseases (%)	18 (21.4)		
Previous malignancies (%)	18 (21.4)		
FH of PDAC (%)	12 (14.3)		

BMI—Body Mass Index; DM—diabetes mellitus; FH—family history; MD-IPMN—main duct intraductal papillary mucinous neoplasm; MRCP—magnetic resonance cholangiopancreatography; PDAC—pancreatic ductal adenocarcinoma.

## Data Availability

All data can be found in patients’ records at Tel Aviv Sourasky Medical Center.

## Appendix A

**Table A1:** Data parameters collected from patients’ records.

Demographic	
Age	
Gender	
Clinical	
IPMN diagnosis date	
MRCP date	
Duration from IPMN diagnosis to first MRCP	
Age (at diagnosis and every MRCP)	
Symptoms	Bloating, nausea, abdominal pain, weight loss, jaundice
Prior medical history	Diabetes mellitus, BMI, dyslipidemia, smoking history, ethanol abuse, liver diseases
	History of pancreatitis or malignancies
Family history	FH of PDAC
Laboratory	Liver enzymes; CA 19-9; CEA; HbA1C
Radiologic	
Cyst number and location, maximal cyst size, MPD diameter, IPMN type, SCA, NET	
Cyst and MPD size progression	Time taken for cyst to progess to 3 and 5 mm in size
Signs of pancreatitis	Acute and chronic pancreatitis
Presence of PDAC	
Extra pancreatic findings	Choledocholithiasis; cholelithiasis; status post cholecystectomy; cholangitis; cholangiocarcinoma; gallbladder polyp
Endoscopic	EUS intrapancreatic findings (cystic lesion, solid mass, worrisome features, high-risk stigmata) PCF analysis including measured levels of amylase, CEA, glucose, and fluid cytology

BMI—Body Mass Index; CA 19-9—carbohydrate-antigen 19-9; CEA—carcinoembryonic antigen; EUS—endoscopic ultrasound; FH—family history; HbA1C—hemoglobin A1C; IPMN—intraductal papillary mucinous neoplasm; MPD—main pancreatic duct; NET—neuroendocrine tumor; PCF—pancreatic cystic fluid (PCF); PDAC—pancreatic ductal adenocarcinoma; SCA—serous cystadenoma.
